# Racial/Ethnic Disparities in Unintentional Fatal and Nonfatal Emergency Medical Services–Attended Opioid Overdoses During the COVID-19 Pandemic in Philadelphia

**DOI:** 10.1001/jamanetworkopen.2020.34878

**Published:** 2021-01-21

**Authors:** Utsha G. Khatri, Lia N. Pizzicato, Kendra Viner, Emily Bobyock, Monica Sun, Zachary F. Meisel, Eugenia C. South

**Affiliations:** 1 National Clinician Scholars Program, Corporal Michael J. Crescenz Veterans Affairs Medical Center, University of Pennsylvania, Philadelphia; 2 Department of Emergency Medicine, Perelman School of Medicine, University of Pennsylvania, Philadelphia; 3 Substance Use Prevention and Harm Reduction Division, Philadelphia Department of Public Health, Philadelphia, Pennsylvania; 4 Philadelphia Fire Department Emergency Medical Services, Philadelphia, Pennsylvania; 5 Center for Emergency Care Research and Policy, Department of Emergency Medicine, Perelman School of Medicine, University of Pennsylvania, Philadelphia; 6 Urban Health Lab, Department of Emergency Medicine, Perelman School of Medicine, University of Pennsylvania, Philadelphia

## Abstract

This cross-sectional study describes the differential associations of the coronavirus disease 2019 (COVID-19) pandemic with opioid-related overdoses among racial/ethnic groups in Philadelphia, Pennsylvania.

## Introduction

Opioid overdoses have increased during the coronavirus disease 2019 (COVID-19) pandemic.^1^ Potential contributors include treatment center closures, physical isolation preventing bystander rescue, mental health stressors, financial instability, and changes to drug supply networks.^2^ Black individuals with opioid use disorder (OUD) may be disproportionately affected, given the racial disparities in COVID-19 morbidity and mortality.^3^ We describe the differential associations of the COVID-19 pandemic with overdoses among racial/ethnic groups in Philadelphia, Pennsylvania.

## Methods

In this cross-sectional study, unintentional fatal opioid-related overdose (FOO) and emergency medical services (EMS)–attended nonfatal opioid-related overdose (NFOO) counts were extracted from deidentified publicly available data from the Philadelphia Department of Public Health’s Substance Use Data Dashboard. The Philadelphia Medical Examiner’s Office was used to identify unintentional opioid overdose deaths. Naloxone administrations by EMS were collected by the Philadelphia Fire Department and used as a proxy for EMS-attended NFOO. Race/ethnicity was classified by the decedent’s family for FOO and by EMS professionals for NFOO. Because this study used aggregated public data, it did not constitute human participant research according to the University of Pennsylvania institutional review board. This study followed the Strengthening the Reporting of Observational Studies in Epidemiology ( STROBE ) reporting guideline for cross-sectional studies.

We analyzed data from January 2019 through June 2020 and compared the mean monthly counts of FOO and NFOO before and after the March 23, 2020, Philadelphia stay-at-home order. Excluding March as a washout period, we compared mean counts in the 3 months after the order (April to June 2020; period C) to 3 months before (December 2019 to February 2020; period B) and the same 3-month period the year prior (April to June 2019; period A). We used a *t* test of equal variance with a 2-sided significance level of *P* < .05. Data were analyzed using Stata/IC version 15.1 (StataCorp).

## Results

Overall, FOO counts were unchanged in period C (monthly mean [SD], 98.0 [6.1]) compared with period A (monthly mean [SD], 94.7 [4.2]). Among non-Hispanic Black individuals, the mean monthly (SD) FOO count increased from 32.0 (3.6) in period A and 30.3 (10.4) in period B to 48.7 (3.1) in period C, representing a 52.1% increase from period A to C and a 60.4% increase period B to C (Table). In contrast, among non-Hispanic White individuals, the mean (SD) counts for periods A to C were 46.3 (2.1), 45.3 (4.2), and 35.3 (7.2), respectively, representing a 23.8% decrease from period A to C and a 22.1% decrease from period B to C. Period C represents the first time in recent history in Philadelphia that the absolute number of deaths was higher among non-Hispanic Black individuals than among non-Hispanic White individuals. Among Hispanic individuals, mean counts decreased from period A to C but increased from period B to C. Similar trends by race were seen for the mean monthly count of EMS naloxone administrations among non-Hispanic Black, non-Hispanic White, and Hispanic individuals (Table and Figure).

**Table.** Mean Monthly Counts of Fatal and Emergency Medical Service–Attended Nonfatal Opioid Overdose by Race/Ethnicity

Race/ethnicity	Period A, April to June2019		Period B, December 2019 to February 2020		Period C, April to June 2020		*P* value	
	Total, No.	Monthly mean (SD)	Total, No.	Monthly mean (SD)	Total, No.	Monthly mean (SD)	Period A and C	Period B and C
**Fatal**								
Non-Hispanic Black	96	32 (3.6)	91	30.3 (10.4)	146	48.7 (3.1)	.004	.04
Non-Hispanic White	139	46.3 (2.1)	136	45.3 (4.2)	106	35.3 (7.2)	.06	.11
Hispanic	49	16.3 (3.1)	38	12.7 (1.5)	42	14 (3.5)	.43	.57
**Nonfatal**								
Non-Hispanic Black	253	84.3 (3.1)	264	88 (9.2)	309	111 (28.8)	.19	.26
Non-Hispanic White	361	120.3 (2.5)	336	112 (5.6)	333	103 (6.9)	.02	.15
Hispanic	127	42.3 (6.0)	113	37.7 (4.9)	124	41.3 (6.4)	.85	.48

**Figure.**
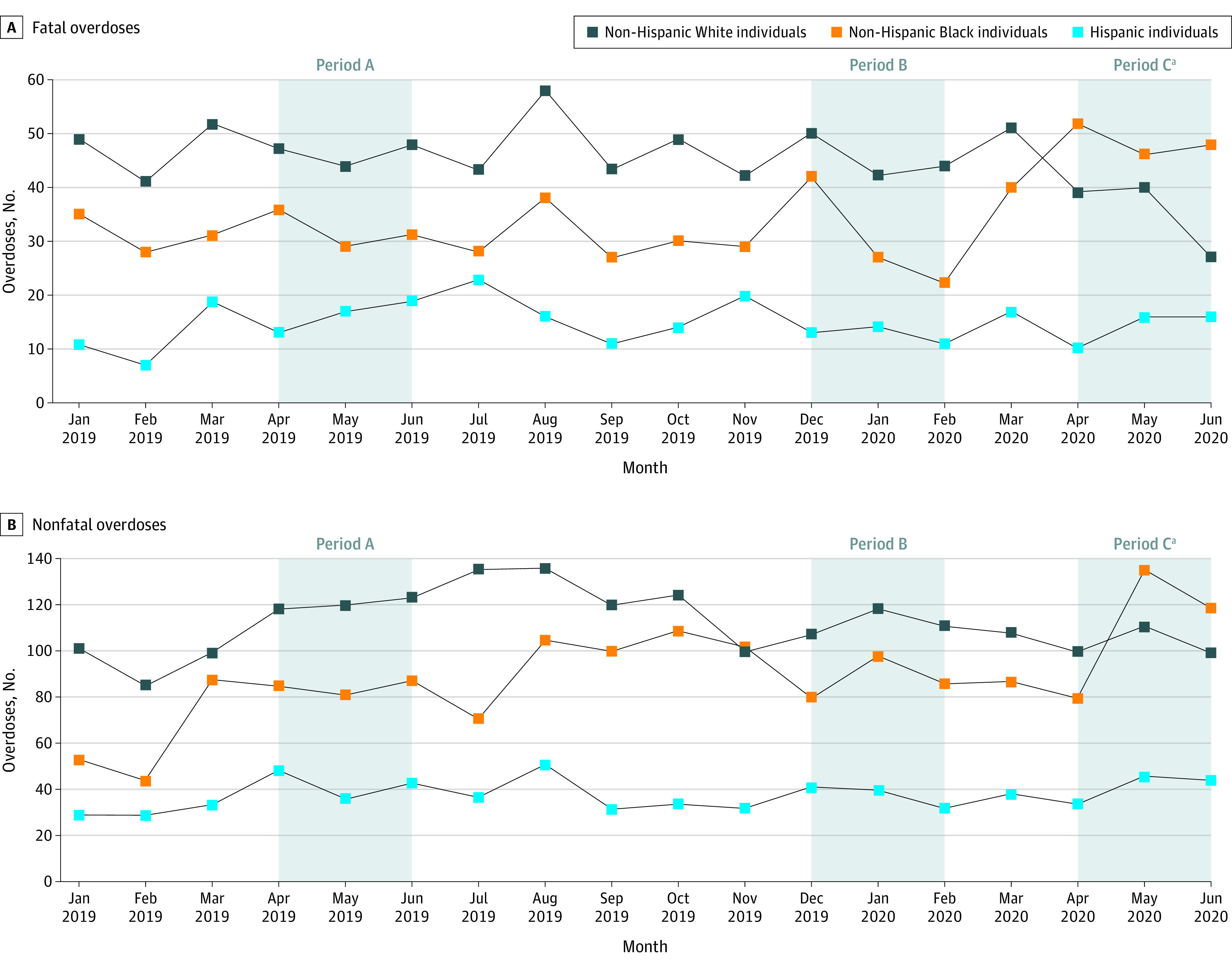
Opioid-Related Fatal and Emergency Medical Services–Attended Nonfatal Overdoses by Race/Ethnicity ^a^Period C indicates period of stay-at-home order, which began on March 23, 2020.

## Discussion

Increasing opioid overdoses during COVID-19 have received national attention in the United States, but there has been little exploration of the differential trends among racial/ethnic groups.^4^ In Philadelphia, COVID-19 was associated with increases in opioid overdose among non-Hispanic Black individuals but decreases among non-Hispanic White individuals. COVID-19 has exacerbated preexisting stressors, social isolation, and economic deprivation disproportionately in Black communities, possibly contributing to increased substance use. The preexisting racial disparities in accessing substance use treatment may also be heightened by COVID-19–related shifts in treatment availability.^5^

Notably, we are limited by our short observation period and inability to determine whether the overdoses were due to inadvertent opioid exposure (eg, fentanyl contamination of stimulants) and primary opioid use vs polysubstance use.^6^ Further analysis with additional months of data to observe random variation is warranted. Disaggregated data from other cities analyzed by race/ethnicity should be prioritized. OUD treatment, harm reduction, and overdose prevention efforts should be immediately targeted to Black and other communities at highest risk during and after the COVID-19 pandemic.

## References

[1] Slavova S, Rock P, Bush HM, Quesinberry D, Walsh SL Signal of increased opioid overdose during COVID-19 from emergency medical services data. Drug Alcohol Depend. 2020;214:108176. doi:10.1016/j.drugalcdep.2020.108176 32717504 PMC7351024

[2] Wang QQ, Kaelber DC, Xu R, Volkow ND COVID-19 risk and outcomes in patients with substance use disorders: analyses from electronic health records in the United States. Mol Psychiatry. 2020. doi:10.1038/s41380-020-00880-7 32929211 PMC7488216

[3] Millett GA, Jones AT, Benkeser D, Assessing differential impacts of COVID-19 on black communities. Ann Epidemiol. 2020;47:37-44. doi:10.1016/j.annepidem.2020.05.003 32419766 PMC7224670

[4] American Medical Association Issue brief: reports of increases in opioid- and other drug-related overdose and other concerns during COVID pandemic. Updated December 9, 2020 Accessed December 11, 2020. https://www.ama-assn.org/system/files/2020-12/issue-brief-increases-in-opioid-related-overdose.pdf

[5] Goedel WC, Shapiro A, Cerdá M, Tsai JW, Hadland SE, Marshall BDL Association of racial/ethnic segregation with treatment capacity for opioid use disorder in counties in the United States. JAMA Netw Open. 2020;3(4):e203711. doi:10.1001/jamanetworkopen.2020.3711 32320038 PMC7177200

[6] Khatri UG, Viner K, Perrone J Lethal fentanyl and cocaine intoxication. N Engl J Med. 2018;379(18):1782-1782. doi:10.1056/NEJMc1809521 30380395

